# Circulating Tumor DNA Detection in the Management of Anti-EGFR Therapy for Advanced Colorectal Cancer

**DOI:** 10.3389/fonc.2019.00170

**Published:** 2019-03-22

**Authors:** Franciele H. Knebel, Fabiana Bettoni, Leonardo G. da Fonseca, Anamaria A. Camargo, Jorge Sabbaga, Denis L. Jardim

**Affiliations:** ^1^Sociedade Beneficente de Senhoras-Hospital Sírio Libanês, São Paulo, Brazil; ^2^Instituto do Câncer do Estado de São Paulo, São Paulo, Brazil; ^3^Ludwig Institute for Cancer Research, São Paulo, Brazil

**Keywords:** liquid biopsies, anti-EGFR therapy, colorectal cancer, drug resistance, monitoring, circulating tumor DNA

## Abstract

**Background:** Anti-EGFR antibodies are a standard care for advanced *KRAS*-wild type colorectal cancers. Circulating tumor DNA (ctDNA) monitoring during therapy can detect emergence of *KRAS* mutant clones and early resistance to therapy.

**Case Presentation:** We describe a 61-years-old man presenting a metastatic and recurrent rectal cancer treated with different chemotherapy regimens. His tumor was *KRAS* wild-type based on tissue analysis and he was treated sequentially with cetuximab-based chemotherapy, chemotherapy alone and panitumumab-based chemotherapy. We performed sequential analysis of ctDNA using droplet digital PCR (ddPCR) and a commercial assay designed for the detection of frequent *KRAS* mutations during his clinical follow-up. Prior to the first cetuximab-based chemotherapy ctDNA analysis demonstrated an absence of *KRAS* mutations. Emergence of *KRAS* mutations in ctDNA occurred ~3 months after treatment initiation and preceded clinical and imaging progression in about 2 months. Fractional abundance of *KRAS* mutation rapidly increased to 70.7% immediately before a chemotherapy alone regimen was initiated. Interestingly, *KRAS* mutation abundance decreased significantly during the first two months of chemotherapy, reaching a fractional abundance of 3.0%, despite minimal clinical benefit with this therapy. Re-challenge with a different anti-EGFR antibody was attempted as later line, but high levels of *KRAS* mutations in ctDNA before therapy correlated with an absence of clinical benefit.

**Conclusions:** The monitoring of resistance mutations in *KRAS* using ctDNA during the treatment of *KRAS* wild-type advanced colorectal cancers can detect the emergence of resistant clones prior to clinical progression. Dynamics of resistant clones may alter during periods on and off anti-EGFR antibodies, detecting window of opportunities for a re-challenge with these therapies.

## Background

Over the past years, substantial advances in cancer genomics established a new era in which information on the genetic background of tumors moved from laboratories to the clinic. For many solid tumors, certain genetic alterations proved to be prognostic, predicting treatment sensitivity, and resistance, especially to targeted therapies. Much of the recent data about somatic genetic alterations were generated based on tissue analysis, obtained in a fixed time point during tumor evolution. Nonetheless, it is well-known that solid tumors change over time and space as a result of clonal evolution, leading to significant intra-tumor genetic heterogeneity (1). In this setting, liquid biopsies are gaining relevance as a tool to capture genetic tumor evolution and intra-tumor genetic heterogeneity more precisely.

Broadly, liquid biopsies consist of diagnostic methods based on the detection of circulating tumor material such as cells, nucleic acids, proteins, and extracellular vesicles in a minimally invasive manner through the sampling of blood or other body fluids. Nevertheless, the detection of circulating tumor DNA (ctDNA) in the plasma is the most clinically useful modality of liquid biopsies due to its high specificity and sensitivity, relative low cost, and straightforward analysis (2). Using ctDNA to characterize genetic alterations is appealing, due to its minimally invasive nature, possibility to represent a background from multiple tumor sites and facility to repeat tests during treatment (3). There are a number of applications for liquid biopsy, but the only clinically approved uses are to monitor treatment response and detect the emergence of drug resistance in a few tumor types (3).

Monoclonal antibodies that specifically target Epidermal Growth Factor Receptor (EGFR) are frequently used as monotherapies or in combination with chemotherapy to treat advanced colorectal cancer. Their benefit in increasing response rate and prolong survival is restricted to patients with *KRAS* wild-type tumors (4, 5). Both drugs currently approved for this setting, namely Cetuximab and Panitumumab, require tissue testing for *KRAS* alterations. More recently, sidedness of the tumor was also implied as a potential predictive factor, as left-side tumors seem to derive a pronounced benefit with anti-EGFR therapies (6).

Despite its efficacy, some patients will not respond to Cetuximab or Panitumumab and the majority of responders will develop resistance at some point during treatment. Since these drugs are commonly used in combination with chemotherapy, it is difficult to discern if disease progression implies resistance to both anti-EGFR and chemotherapy or just one of these agents. Some authors suggested that resistance can occur only with the chemotherapy component and, in this scenario, maintaining the EGFR blockade while changing the chemotherapy backbone could be a strategy (7). On the other hand, it has been demonstrated that, in patients developing resistance to anti-EGFR therapies, a period free of therapy targeting this receptor could re-sensitize tumors by reducing the clonal selection pressure. In this setting, re-challenge with an anti-EGFR therapy would be able to produce further tumor regression (8).

Resistance to anti-EGFR antibodies is mainly driven by the emergence of mutations in certain genes during treatment, especially in *KRAS, NRAS, BRAF*, and *EGFR* (9). Recent studies suggested that some of these alterations may be multi-clonal, and, thus, associated with intra and/or inter-lesions heterogeneity (10). In this context, longitudinally monitoring the landscape of genetic alterations during treatment with anti-EGFR antibodies could help to detect the emergence and dynamics of the mutations associated with resistance and guide the decision-making process when choosing between anti-EGFR therapy continuation vs. re-challenge (11).

Here, we describe a case of a patient with advanced *KRAS* wild-type colorectal cancer treated with anti-EGFR therapy in combination with chemotherapy that was monitored with sequential analysis of ctDNA using droplet digital PCR (ddPCR) and a commercial assay designed for the detection of frequent *KRAS* mutations (*KRAS* G12/G13 Screening Kit—BioRad). We evaluated clinical response during sequential systemic therapies including two different anti-EGFR antibodies, along with dynamics of *KRAS* status in ctDNA.

## Case Presentation

KLM, a North American white man, was 61 years old in August 2010 when he was diagnosed with a distal rectal cancer, clinically staged as T3N1M0. His initial therapeutic approach included neoadjuvant radio/chemotherapy followed by close surveillance, since digital rectal examination, proctoscopy and pelvic MRI, at the end of treatment, were normal.

In September 2011, an increase in serum levels of Carcinoembryonic Antigen (CEA) was noted. A local relapse and a 3 cm lesion in liver segment VIII were simultaneously diagnosed. Some suspicious, but undetermined, small lung nodules were also observed at that time. Patient was initially submitted to a full-thickness transanal excision and then to neoadjuvant (perioperative) chemotherapy with FOLFOX followed by hepatectomy and adjuvant FOLFOX. Intensive proctologic follow up was still maintained. Molecular analyses of the tumor obtained from liver metastasis showed *KRA*S and *BRAF* wild-type status.

On December 2013, lung metastases became clear and first line chemotherapy with FOLFIRI/bevacizumab was initiated. Patient was treated with this regimen until June 2015, when new hepatic lesions were detected and chemotherapy changed to irinotecan with cetuximab (CPT11/CTX). At that time, the patient agreed by written consent to have his blood periodically collected for molecular testing. He was informed that results of these tests would be, however, kept unrevealed until at least the end of treatment with anti-EGFR.

Blood samples were collected periodically from June 2015 until April 2017 and the emergence and dynamics of KRAS mutations in ctDNA was monitored using ddPCR as previously described (12). Briefly, 15 ml of blood were collected using tubes containing EDTA. Plasma was separated from blood by centrifugation within 2 h after collection and plasma was stored at −80°C. Cell free DNA was isolated using the QIAamp MinElute Virus Vacuum Kit and stored at −80°C. We used a RNase P Copy Number Reference Assay to determine the total amount of DNA in plasma samples and a commercial assay designed for the detection of frequent KRAS mutations (KRAS Screening Kit BioRad – G12V, G12D, G12A, G12C, G12R, G12S e G13D). A total of 3000–3500 genome equivalents were analyzed per reaction for a detection sensitivity between 0.1 and 0.5%. ddPCR was performed on the QX200 Droplet Digital PCR System and data were analyzed using QuantaSoft software. ctDNA detection results are presented as fractional abundance (proportion of the mutant allele in total circulating DNA) for comparison between different time points.

**Figure 1:**
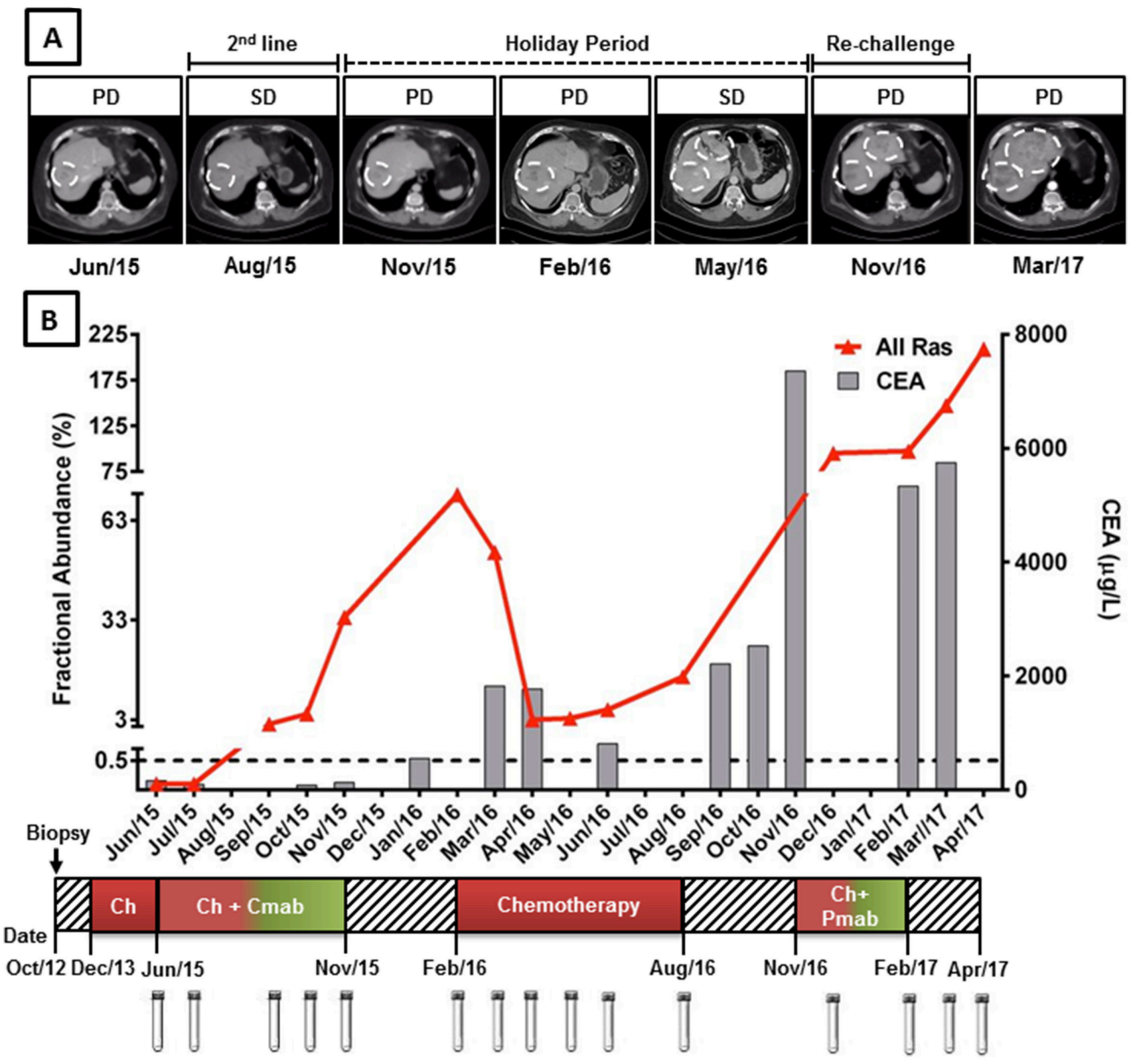
*KRAS* G12/13 longitudinal monitoring and clinical response to anti-EGFR mAb plus chemotherapy in cell-free circulating DNA of a mCRC patient. **(A)** Computed tomography (CT) images of liver metastasis during anti-EGFR mAb treatment, holyday and mAb re-challenge. **(B)** Red line indicates the fractional abundance of *KRAS* mutation (percentage of mutated alleles in a total DNA background) detected in ctDNA at treatment course (X-axis) by ddPCR. Approximately 3,000 haploid genome equivalents were analyzed for each time point (*KRAS* limit of detection 0.5%). Gray columns represent CEA levels. *KRAS* mutation detection was able to anticipate clinical progression, preceding CEA elevation. **(A,B)** *KRAS* levels decreased during anti-EGFR holyday and chemotherapy switch coinciding with stable disease. High levels of *KRAS* mutations after chemotherapy progression were able to predict poor response to anti-EGFR re-challenge. Cmab, cetuximab; Pmab, panitumumab; Chemo, chemotherapy; SD, stable disease; PD, progressive disease.

At the beginning of CPT11/CTX treatment, in June 2015, blood samples were negative for the presence of *KRAS* mutations (Figure 1). First evaluation of response was performed after 6 cycles in August 2015 and showed stable disease by RECIST, with an expressive decline in serum CEA (from 162 to 80 μg/L). Treatment was maintained until clinical progression was observed in November 2015 at expenses of significant skin and gastrointestinal toxicities. *KRAS* mutations became detectable in September 2015, anticipating clinical disease progression, and raised considerably over the next 2 months reaching a fractional abundance of 33.8% in November 2015 (Figure 1).

From November 2015 to February 2016 patient remained in a “drug holiday period,” during which no chemotherapy was administered. Rapid CEA elevation and CT scans denoting progression in pulmonary and liver metastasis have induced a new treatment to begin. A significant increase in *KRAS* mutation fractional abundance was also observed during this period (from 33.8 to 70.7% in February 2016) (Figure 1). He was then re-challenged with FOLFOX, achieving again an initial clinical benefit (small reduction in tumor sizes and CEA response) followed by progression of disease on August 2016. *KRAS* mutation abundance decreased significantly during the first two months of FOLFOX treatment, reaching a fractional abundance of 3.0% in April 2016. However, *KRAS* mutation abundance started to increase steadily thereafter, anticipating, once more, clinical disease progression (Figure 1).

A fourth line of palliative chemotherapy, combining irinotecan with panitumumab was also tried from November 2016 to February 2017 without success. High levels of *KRAS* mutations in ctDNA were detected in December 2016, remaining relatively stable until February 2017 and anticipating poor response to palliative treatment and disease progression. The best supportive care was offered up to patient's death in June 2017. A significant increase in *KRAS* mutation abundance in ctDNA was observed after the interruption of palliative treatment (Figure 1).

## Discussion

Monoclonal antibodies anti-EGFR in combination with chemotherapy is one of the standard treatments for RAS wild-type metastatic colorectal cancer. However, many RAS wild type patients do not respond to anti-EGFR therapies and even those who initially respond to therapy will ultimately progress, at least in part, because of the emergence of *KRAS* mutations.

Retrospective studies including a small number of patients with mCRC patients treated with anti-EGFR therapy have shown that ctDNA analysis in plasma samples can detect acquired mutations in *KRAS* leading to therapy resistance (8, 13, 14). In this scenario, longitudinal monitoring of *KRAS* status using liquid biopsies during anti-EGFR therapy may allow the early detection of acquired resistance and guide clinical decision to switch to a subsequent line of therapy, increasing the likelihood of the patient to derive maximal benefit from sequential therapy. Longitudinal monitoring of *KRAS* status may be particularly useful to guide the decision-making progress when choosing between anti-EGFR therapy continuation vs. re-challenge (7).

Although the use of liquid biopsies to monitor cancer patients is a technically feasible and affordable procedure, our current knowledge of ctDNA detection in cancer patients must be expanded before liquid biopsies can be routinely implemented into clinical practice (15). In this case study, liquid biopsies were used to monitor the emergence and dynamics of *KRAS* mutations in a patient with advanced *KRAS* wild-type colorectal cancer treated with anti-EGFR therapy in combination with chemotherapy. It is important to highlight that ctDNA analysis was performed retrospectively and that ctDNA detection results did not influence the therapeutic decisions for this patient.

The clinical decision to select an anti-EGFR as part of the therapy is driven by a *KRAS* wild-type mutational testing in the tissue and a variety of clinical factors. In this case study, Cetuximab was associated to CPT11 as a second line therapy. Comparative studies showed no overall survival difference between anti-EGFR and bevacizumab-based regimens (which was the choice for initial palliative therapy for this patient) as a first line therapy for *KRAS* wild-type colorectal cancers (16, 17). For this patient, *KRAS* testing was performed in a liver metastasis prior to initiation of first-line therapy. In accordance with tissue genotyping, our ctDNA analysis before initiation of the anti-EGFR therapy demonstrated an absence of *KRAS* mutations. Prior studies established over 90% agreement between plasma and tissue *KRAS* status, (18, 19) which reinforces that, in fact, this patient was wild-type by current clinical guidelines. Interestingly, higher sensitive techniques for *KRAS* plasma detection may identify a higher number of patients with mutations that were actually negative by tissue testing (20). It is unknown, however, if such a finding may alter initial clinical management of patients, as survival for patients receiving first line Cetuximab-based therapy is similar compared to patients submitted to tissue and plasma biopsies (21).

Our results corroborate previous studies that have shown that ctDNA detection can be used to track the emergence of tumor resistant subclones during anti-EGFR therapy, allowing early detection of drug resistance and disease progression. Marked increases in *KRAS* mutation abundance was detected in blood samples from our patient 2 months before clinical progression of the disease after the first exposure to anti-EGFR therapy. Others authors reported that the allelic frequency of mutations in plasma from CRC patients (including *KRAS*) may be an indicator of response or resistance to systemic therapy (8, 13, 14). Thus, ctDNA can be helpful for monitoring response, also considering that up to 30% of patients with CRC do not show alterations in CEA blood levels (22). Our case also illustrates that elevation in the allelic frequency of *KRAS* mutations also preceded CEA elevation.

Finally, our results also suggest that ctDNA analysis can be efficiently used to monitor the dynamics of *KRAS* mutated resistant clones during systemic treatment of mCRC and to identify patients eligible for anti-EGFR therapy continuation or re-challenge. Interestingly, marked decreases in *KRAS* mutation abundance were not observed in our patient immediately after anti-EGFR was withdrawn (November 2015 and February 2017), but were detected readily after the chemotherapy switch. Recent data demonstrated that *KRAS* mutant clones might decline after stopping an anti-EGFR therapy, similarly to *KRAS* allelic fraction in plasma (8, 23). This finding suggests that the RAS-resistant phenotype may be reversible, leading to new opportunities to use anti-EGFR therapies. On the other hand, high levels of *KRAS* mutations in ctDNA were able to predict poor response to anti-EGFR re-challenge, as illustrated in our case report. It is plausible to hypothesize that re-challenge with an anti-EGFR therapy might be better offered to patients with no resistance mutations on ctDNA.

## Conclusions

In conclusion, our results demonstrate that colorectal tumor genomes adapt dynamically to intermittent anti-EGFR treatment and indicates that liquid biopsy is a promising tool to monitor acquired resistance to anti-EGFR therapy and guide second line treatment strategies.

## Data Availability

All datasets generated for this study are included in the manuscript and/or the supplementary files.

## Ethics Statement

Patient has signed an informed consent form to participate in the study. This study was approved under number # 2015–22 by the ethics committee of the Instituto de Ensino e Pesquisa do Hospital Sírio Libanês.

## Author Contributions

FK and FB carried out the ctDNA analysis. JS was responsible for patient clinical management. FK, FB, AC, and JS conceived and designed the analysis. All authors analyzed the data, wrote and revised the manuscript, and read and approved the final version for submission.

### Conflict of Interest Statement

DJ received honoraria from Janssen-Cilag, Bristol-Myers Squibb, Roche, and MSD; travel expenses by Bristol-Myers Squibb and Janssen-Cilag. JS received honoraria from Bayer, Merck Serono, and MSD; travel expenses by Bayer. The remaining authors declare that the research was conducted in the absence of any commercial or financial relationships that could be construed as a potential conflict of interest.

## Abbreviations

CEA: carcinoembryonic antigen
ctDNA: circulating tumor DNA
cfDNA: cell free DNA
ddPCR: digital droplet PCR
MRI: magnetic resonance imaging
RECIST: response evaluation criteria in solid tumors
CT: computed tomography
mCRC: metastatic colorectal cancer.
